# Anticancer drug-loaded multifunctional nanoparticles to enhance the chemotherapeutic efficacy in lung cancer metastasis

**DOI:** 10.1186/s12951-014-0037-5

**Published:** 2014-09-30

**Authors:** Jian-Ting Long, Tuck-yun Cheang, Shu-Yu Zhuo, Rui-Fang Zeng, Qiang-Sheng Dai, He-Ping Li, Shi Fang

**Affiliations:** Department of Medicinal Oncology, The First Affiliated Hospital, SUN Yat-Sen University, Guangzhou, 510080 China; Department of Vascular Surgery, The First Affiliated Hospital, SUN Yat-Sen University, Guangzhou, 510080 China; Department of Clinical Nutrition, The First Affiliated Hospital, SUN Yat-Sen University, No. 58, cprZhongshan 2nd Road, Guangzhou, Guangdong 510080 China

**Keywords:** Doxorubicin, EGF, EGFR, Gelatin nanoparticles, Lung cancer, Inhalation, Ligand targeting, Controlled release

## Abstract

**Background:**

Inhalation of chemotherapeutic drugs directly into the lungs augments the drug exposure to lung cancers. The inhalation of free drugs however results in over exposure and causes severe adverse effect to normal cells. In the present study, epidermal growth factor (EGF)-modified gelatin nanoparticles (EGNP) was developed to administer doxorubicin (DOX) to lung cancers.

**Results:**

The EGNP released DOX in a sustained manner and effectively internalized in EGFR overexpressing A549 and H226 lung cancer cells via a receptor-mediated endocytosis. In vitro cytotoxicity assay showed that EGNP effectively inhibited the growth of A549 and H226 cells in a dose-dependent manner. In vivo biocompatibility study showed that both GNP and EGNP did not activate the inflammatory response and had a low propensity to cause immune response. Additionally, EGNP maintained a high therapeutic concentration in lungs throughout up to 24 h comparing to that of free drug and GNP, implying the effect of ligand-targeted tumor delivery. Mice treated with EGNP remarkably suppressed the tumor growth (~90% tumor inhibition) with 100% mice survival rate. Furthermore, inhalation of EGNP resulted in elevated levels of cleaved caspase-3 (apoptotic marker), while MMP-9 level significantly reduced comparing to that of control group.

**Conclusions:**

Overall, results suggest that EGF surface-modified nanocarriers could be delivered to lungs via inhalation and controlled delivery of drugs in the lungs will greatly improve the therapeutic options in lung cancer therapy. This ligand-targeted nanoparticulate system could be promising for the lung cancer treatment.

## Background

Lung cancer is one of the leading causes of cancer mortality in developed and underdeveloped countries, and responsible for 25% of death due to cancer [1]. The death rate due to lung cancer precedes the number of death due to colorectal, breast, prostate and pancreatic cancers [2]. Lung cancer can be broadly divided into small cell lung cancer and non-small cell lung cancer (NSCLC) with latter constitutes nearly 80% of lung cancer mortality. Besides, these cancers metastasizes by migrating to blood circulation, from where it invade slowly into pulmonary capillaries and surrounding tissues and regenerate secondary cancer sites [3]. Presently, conventional treatment modalities such as surgical resection, radiotherapy and chemotherapy are standard treatment regimen to for lung cancers. However, surgical removal of cancerous tissues is highly difficult in many cases; radiotherapy damages the normal healthy cells surrounding the cancer cells, and chemotherapy requiring high dose level of individual drugs to treat lung carcinoma [4]. Specifically, chemotherapy which is popularly employed to treat cancer is rarely successful due to the less amount of drug available in lung tissue even if administered at high doses. Most of the times, chemotherapeutic drugs act on the normal cells causing a severe dose-limiting adverse side-effects, and remains far from satisfactory [5]. Therefore, a strong need to develop a therapeutic approach that can increase the efficiency and minimize the adverse effects continuously persists.

In this regard, a therapeutic strategy wherein anticancer drugs are directly delivered into the lungs would be of significant importance. Inhaled delivery system is reported to improve the local drug concentration in lung tissues comparing to that of systemically administered drugs [6-8]. The lung offers high absorption and surface area (100 m^2^) such that drug can be rapidly absorbed, rapid onset of action, high local concentration, and most importantly it can minimize the systemic absorption of drug and so are the reduced side-effects [9]. Although, administration of free drug (via inhalation) enhanced the therapeutic efficacy, however is associated with unwanted toxicity to normal cells in the region. Therefore, development of safe delivery system than can release the therapeutic moiety in a sustained manner to cancer cells while at the same limit its exposure to healthy cells has stimulated a great interest among researchers [10,11]. In this regard, novel drug delivery system such as polymeric or lipid nanoparticles has been successfully developed as part of biopharmaceutical and formulation advancements [12,13]. Moreover, application of nanoparticles to tumors can be further improved by conjugating some active targeting moiety. The active targeting ligand allows the more specific recognition, preferential interaction with cancer cells, and high accumulation of drug in cancer tissues with low/limited presence in normal cells, thereby enhancing the clinical treatment of solid tumors [14,15]. In the present study, gelatin nanoparticles (GNP) was prepared and conjugated with epidermal growth factor (EGF) as an active targeting moiety. EGF was selected as a targeting ligand due to the overexpression of epidermal growth factor receptor (EGFR) in most of the tumors, specifically on NSCLC [16]. Doxorubicin (DOX), an anthracycline antibiotic, was selected due to its broad spectrum of action against lung cancers [17].

Present study aimed at treating lung cancers by the inhalation of DOX-loaded EGF-conjugated gelatin nanoparticles (EGNP) by nebulizer technique. We hypothesized that pulmonary delivery of EGNP will allow better intratumoral administration of DOX-loaded nanotherapeutics resulting in superior anticancer efficacy. For this purpose, we developed gelatin nanoparticles (GNP) which was surface modified with NeutrAvidin-biotinylated epidermal growth factor (bEGF) to facilitate EGFR-mediated endocytosis in tumor cells (NSCLC). Various physicochemical and biological experiments were performed to prove our hypothesis. Additionally, in vivo studies have been conducted in A-549 cancer cell bearing nude mice to systemically evaluate the antitumor efficacy.

## Results and discussion

### Formulation and characterization of EGF-conjugated gelatin nanoparticles

**Figure 1:**
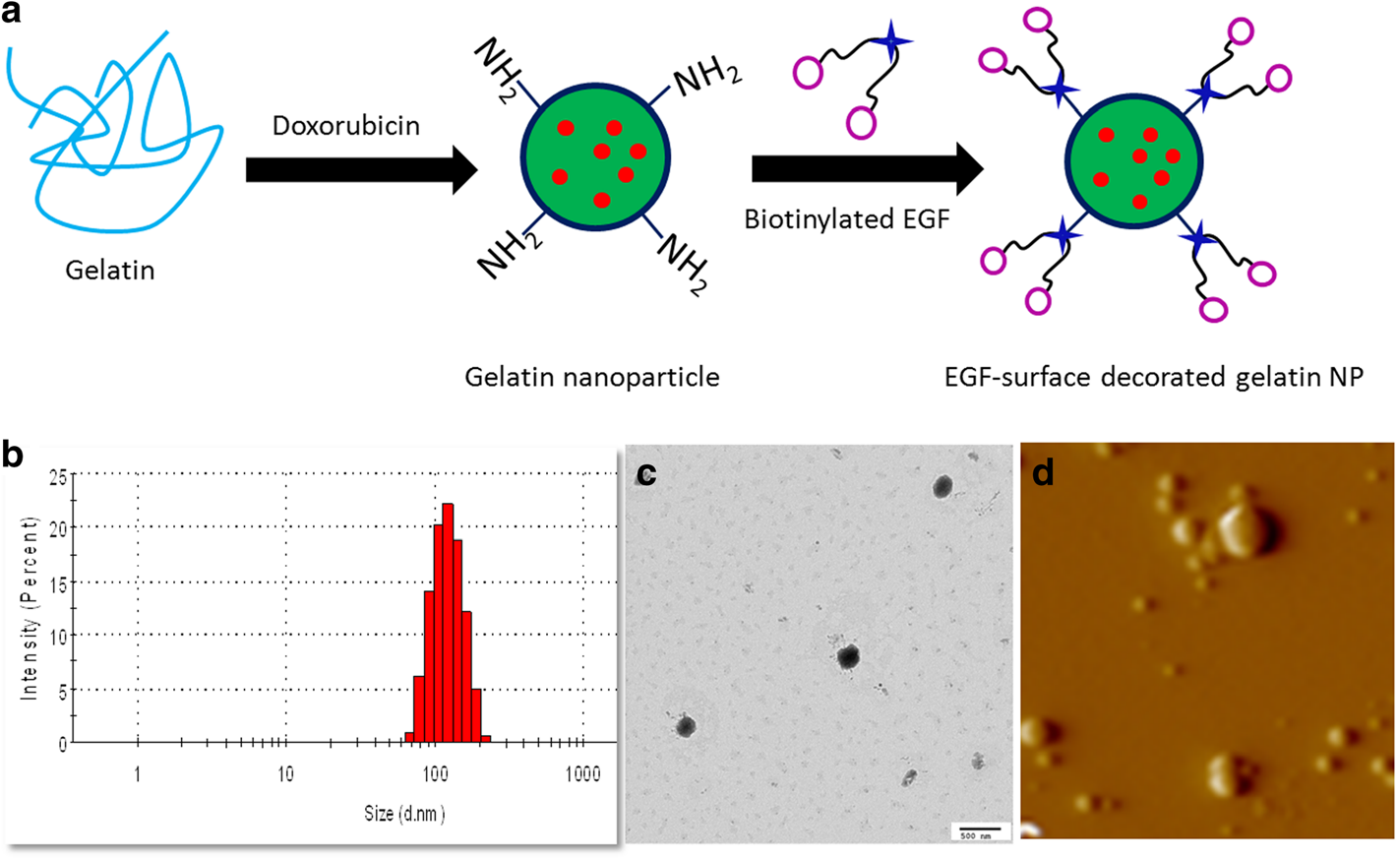
**Physicochemical characterization of nanoparticles. (a)** Schematic presentation of EGF-conjugated gelatin nanoparticles (GNP). DOX-loaded gelatin nanoparticle was formed which was then surface modified with NeutrAvidin-biotinylated-EGF complex to result EGF-conjugated gelatin nanoparticles (EGNP) **(b)** Particle size distribution of EGNP **(c)** TEM mage of EGNP **(d)** AFM image of EGNP.

Inhalation of anticancer drugs directly into the lungs could result in high localization of therapeutic moiety and can greatly improve the chemotherapy against sensitive and resistant lung cancers. However, direct exposure of free drug usually associated with severe side-effects in non-cancerous health cells of the body [18]. Therefore, focus was made to specifically kill the cancer cells while sparing the normal cells. Recently, increased attention has been paid for the development of inhalable drug delivery systems which can release the chemotherapeutic drug in a sustained manner in the local region. This approach was further improved by conjugating an active targeting ligand to the delivery vehicle which will be specific only to the cancer cells in a receptor-mediated pathway. Such system is expected to release its therapeutic load in the lungs either within the tumor microenvironment or within the cancer cells [16]. To this end, GNP was prepared by a two-step desolvation method by evaporating the organic solvent. The DOX-loaded nanoparticle was then surface modified NeutrAvidin-biotinylated-EGF conjugation (EGNP) (Figure 1a). The natural gelatin is abundant in free amino (−NH2) group which was employed to chemically conjugate with EGF. For this first, free amino group of gelatin was converted to thiol group by reacting with 2-iminothiolane. This thiol was then reacted with activated NeutAvidin of EGF chain.

The particle size of nanoparticles plays an important role in the intratumoral distribution and cellular internalization. It has been reported that smaller nanoparticles have greater access to the tumor region in the lungs. In the present study, dynamic light scattering (DLS) analysis showed a nanosized particle for EGNP (~120 nm) with a narrow dispersity index (Figure 1b). The particle size was again confirmed with transmission electron microscope (TEM) and atomic force microscopy (AFM). TEM image showed a black spherical particle which was uniformly dispersed on the copper-coated carbon grid. Moreover, the TEM size was consistent with the DLS measurement and no discrepancy was observed in the dried state (Figure 1c). The surface topography was further confirmed by the AFM analysis which exhibited a flat circular particle on the mica surface (Figure 1d). The particles were distinguishly separate from one another and maintained their size and shape. The particles are flatted on the mica surface owing to the strong physical interaction between each other. Furthermore, entrapment efficiency was observed to be more than 90% with an effective loading capacity of ~20%.

### In vitro drug release

**Figure 2:**
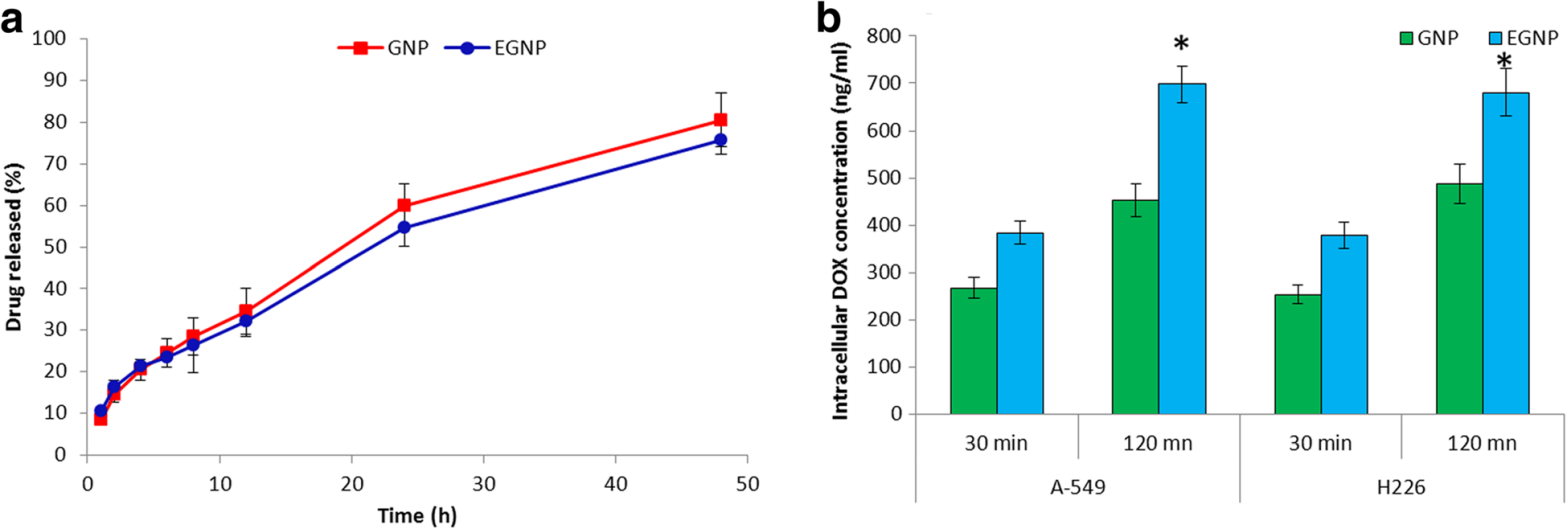
**Drug release and cellular uptake study. (a)** In vitro release study of DOX from GNP and EGNP. The release study was performed in phosphate buffered saline (pH 7.4) at 37°C. The samples were collected at specified time points for up to 48 h and analysed by spectrophotometer. **(b)** Cellular uptake efficiency of GNP and EGNP in A549 and H226 cancer cells. Cells were incubated with 20 μg/ml (DOX equivalent) for 30 and 120 min and analysed using HPLC method.*p < 0.05 is the statistical difference between EGNP and GNP.

The release profile of DOX from GNP and EGNP was performed in phosphate buffered saline (pH 7.4). As can be seen (Figure 2a), an initial burst release of ~10% was observed from both the nanoparticulate system, followed by a sustained release of drug (~70%) towards the end of 48 h of study period. Importantly, no difference in release pattern were observed between GNP and EGNP indicating that substitution or presence of EGF moiety on the nanoparticle surface did not deter the drug release from the delivery system. The release kinetics was confirmed by fitting into four mathematical models including zero order, first order, Higuchi and Korsmeyer–Peppas model. Of all this model, Higuchi model (r = 0.998) best fitted the release profile indicating a diffusion based release. To describe drug release mechanism more precisely, semi-empirical formula, called the Korsmeyer-Peppas power law was employed. The exponent value of 0–0.4 indicates the fickian diffusion while 0.4-0.89 indicates the non-fickian release characteristics. In the present study, an ‘n’ value of 0.68 clearly suggests a non-fickian mode of release and diffusion and erosion being the main mechanism of action. Such a controlled release system by which the drug will be available to the tumor tissue in a constant manner is of significant importance.

### Intracellular drug uptake

In order to DOX within the cancer cells, the nanoparticles have to be internalized into the cells wherein the drug is released and available for its pharmacological action. In this study, we have performed the cellular uptake in two different lung cancer cell lines A549 and H226. The final concentration of DOX exposed to cells was 20 μg/ml and cell uptake was observed at 30 and 120 min. GNP and EGNP were readily internalized in both the cell lines in a time dependent manner (Figure 2b). EGNP showed 2-fold higher cellular internalization than comparing to GNP in A549 and H226 cell lines. The remarkable uptake of EGNP was attributed to the receptor-mediated endocytosis. The presence of EGF on the nanoparticle surface got specific affinity towards EGFR receptors which are overexpressed in both the lung cancer cell lines [19]. On the other hand GNP follows a non-specific adsorption or non-specific interaction with the cells resulting in markedly lower uptake by comparison to ligand-conjugated nanocarriers [20]. Therefore, it can be expected that EGF guidance will allow the specific interaction of carrier to the cancer cells leading to enhance antitumor response.

### In vitro cytotoxicity analysis

**Figure 3:**
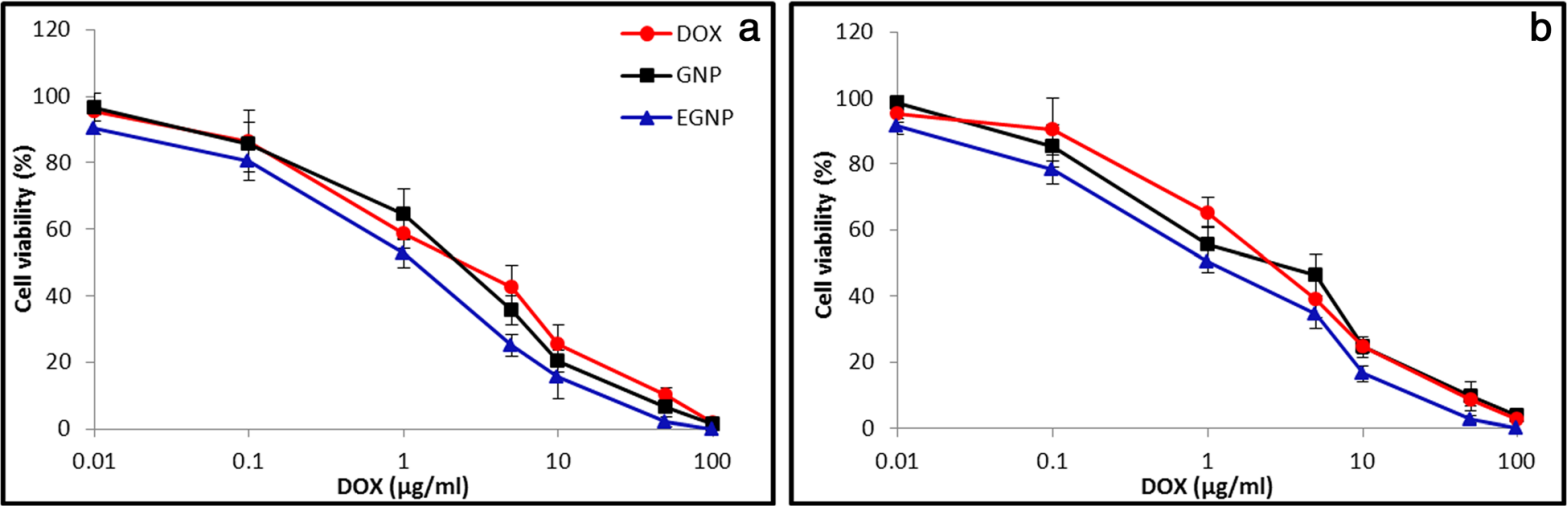
**Cytotoxicity analysis of nanoparticles.** In vitro cytotoxicity analysis of free DOX, GNP, and EGNP on **(a)** A549, **(b)** H226 cell lines. Cell viability assay was performed by MTT assay. The cells were seeded at a density of 10000 cells per well and incubated overnight prior to the exposure of respective formulations for 24 h at 37°C. The IC50values were calculated by GraphPad Prism using nonlinear regression analysis.

The in vitro cytotoxicity analysis was carried out in A549 and H226 cell lines (MTT assay). Free DOX, GNP and EGNP was exposed to these cell lines and incubated for 24 h and dose–response curve was developed. As can be seen, all the therapeutic formulations showed a concentration-dependent cytotoxicity in A549 and H226 cancer cell lines (Figure 3a,b). The difference in cytotoxicity between these cell lines was attributed to the different biological origin and presence of different level of drug-resisting receptors (such as p-glycoprotein). Specifically, EGNP showed the maximum antiproliferative effect in these cell lines comparing to that of free DOX and GNP. Consistently, IC50 value of EGNP was significantly lower than other two formulations in these cell lines (0.56 μg/ml and 0.47 μg/ml in A549 and H226 cells). The enhanced cell killing effect of EGNP was in accordance with the enhanced cellular uptake for this group. The nanoparticles after the endocytosis uptake will immediately escape the endocytic compartments and reach the acidic lysosome wherein drug will be released continuously which diffuse into the nuclear core complex [21]. DOX acts by binding with the topo-isomerase enzyme, intercalate the DNA, and arrest the cell growth. It can be expected that at longer incubation (24 h) time, sub-G1 phase population might decrease, while the G2/M phase population might be increased. Simultaneously, apoptotic and necrotic cells and cell debris can be detected as a “sub-G1” population [22]. It can be safely expected that overexpression of EGFR in cancer cells might provoked the preferential uptake of nanoparticles that resulted in the superior anticancer activity.

### In vivo biocompatibility of nanoparticles

**Figure 4:**
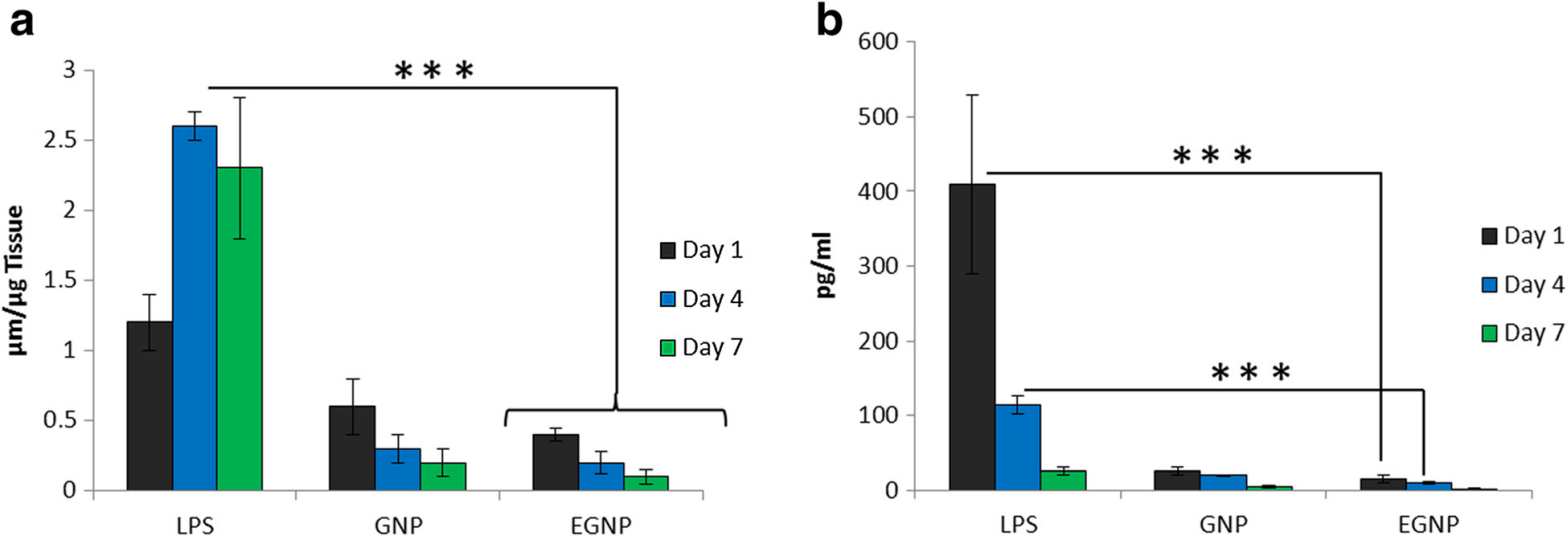
**In vivo biocompatibility of nanoparticles** **In vivo biocompatibility of nanoparticles. (a)** Glutathione level estimation in lung tissue collected from mice treated with single dose inhalation. Lungs were harvested at day 1, 4, and 7 post treatments with GNP, EGNP, and lipopolysaccharide. **(b)** IL-6 level estimation in lung tissue collected from mice treated with single dose inhalation. At respective days BAL fluid was collected and processed. ***p < 0.0001 is the statistical difference between EGNP and control (LPS).

An ideal delivery system is the one which can target the anticancer drugs to the tumor interstitial spaces while being non-toxic to normal cells. In this regard, biocompatibility of GNP and EGNP was studied in mouse model. For this purpose, blank NPs were administered by inhalation then mice were sacrificed at respective time intervals (day 1, 4, and 7). Firstly, oxidative stress was evaluated by assaying the total glutathione levels in the homogenized lung samples. LPS was used as a positive control which is known to induce the oxidative stress in the lungs. As is seen, glutathione levels were remarkably increased in all the formulations at day 1, possibly attributed to the invasive nature of nanoparticles administration (Figure 4a). However, in case of GNP and EGNP, glutathione level continues to decrease until day 7. This was in contrast to the LPS treated mice group which showed a remarkable increase in the biomarker level. Subsequently, IL-6 levels in bronchoalveolar lavage (BAL) fluid samples were estimated at respective days as mentioned above. As expected, IL-6 levels were significantly high in LPS treated group than comparing to either GNP or EGNP treated groups (Figure 4b). IL-6 acts as both a pro-inflammatory and anti-inflammatory cytokine, and is secreted by T-cells and macrophages to stimulate an immune response during infection and after tissue trauma was also investigated as a marker of immune response [23]. Therefore, results indicate that either GNP or EGNP did not activate the inflammatory response and had a low propensity to cause immune response even when delivered directly into the lung tissues. Generally, exposure of inhaled nanoparticles to lungs leads to a range of inflammation and macrophages reactions and activation of immune system. Present study however showed a relatively low toxicity response of the lungs to pulmonary nanoparticles indicating that the delivery carrier is highly biodegradable and biocompatible. Such a biocompatible and biodegradable delivery system could be significant importance in the local delivery to lungs.

### DOX concentration following pulmonary administration

**Figure 5:**
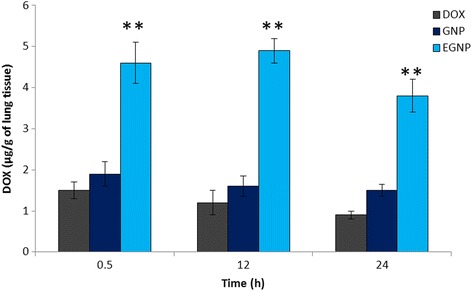
**DOX concentration in lungs following a single dose inhalation of free DOX, GNP, and EGNP.** The DOX concentration in lungs was determined after 0.5, 12, and 24h dose administration. The DOX from EGNP was significantly (p < 0.001) higher than GNP and free DOX. **p < 0.001 is the statistical difference between EGNP and GNP.

For the effective anticancer therapy, a constant exposure of DOX from nanoparticle is desired. For this purpose, DOX concentration in lungs following pulmonary administration was evaluated. As can be seen (Figure 5), approximately ~1.5 μg/g of lung was observed following the administration of free drug, while GNP did not increase the DOX concentration beyond a limit. In contrast, EGNP significantly maintained a high DOX concentration (~5 μg/g of lung) in the lungs following the pulmonary administration in 30 min. There was literally no improvement in the DOX level from GNP and free drug even after 12 h of inhalation, while EGNP still maintained higher drug concentration indicating that EGF surface modification facilitated the accumulation of drug in the tumor region. Surprisingly, EGNP treated group remarkably maintained a higher lung concentration even after 24 h of administration of formulations. It has been reported that NSCLC cells overexpress the EGFR receptor which got specific affinity towards the EGF moiety. From this finding, it would be safe to confirm that GNP/EGNP which was nebulized into minidroplet of a suitable mass and aerodynamic diameter could easily escape the mucociliary clearance to lower respiration track. This tendency agrees with our intratumoral distribution pattern which showed markedly higher fluorescence intensity. Data suggests that the DOX liberated from the gelatin nanoparticles in a controlled and sustained manner such that a constant level of drug is exposed to cancer cells. Results further indicate that cancer cells overexpressed with EGFR could be effectively recognized by EGF modified nanoparticles leading a high cellular accumulation in cancerous lungs. Besides, high accumulation in the lung cancers will eventually avoid the systemic circulation that will result in severe drug-related adverse effects.

### In vivo anticancer efficacy

**Figure 6:**
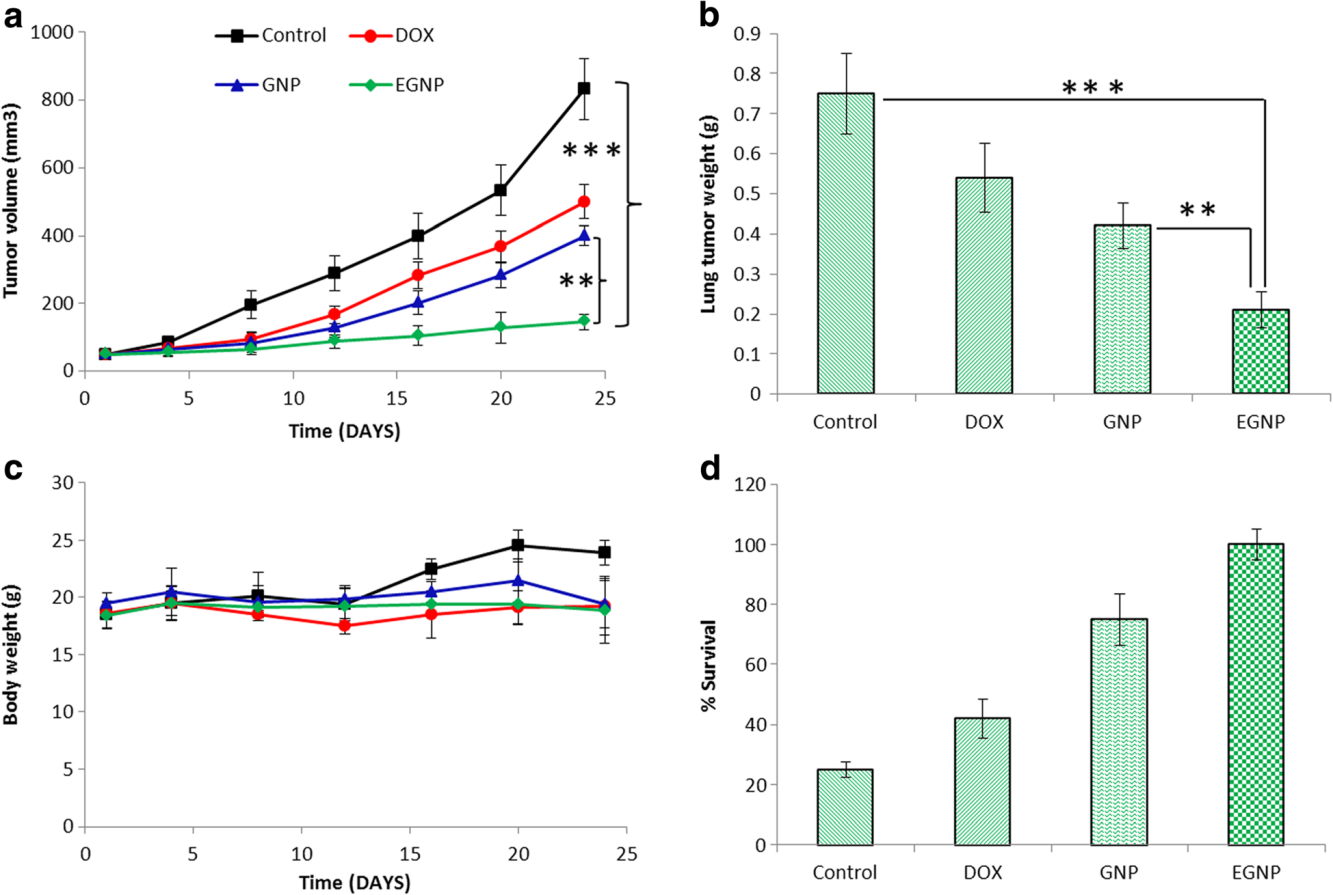
**In vivo antitumor efficacy study in A549 cells bearing xenograft tumor model after intranasal treatment of free DOX, GNP, and EGNP. (a)** In vivo anti-tumor effects **(b)** lung tumor mass **(c)** body weight changes **(d)** survival rate of mice. Each data point was represented as mean ± sd (n = 4). ***p < 0.0001 is the statistical difference between EGNP and control. **p < 0.001 is the statistical difference between EGNP and GNP.

The antitumor efficacy of GNP and EGNP was evaluated in A549 tumor cell bearing nude mice. All the formulations were administered 4 times during first 2-weeks after the onset of experiment at a dose of 5 mg/kg. As shown in Figure 6a, the tumors in untreated controlled group grew linearly, rapidly, and attained maximum tumor volume (~900 mm^3^) at the end of study period. In contrast, mice treated with formulations contracted the growth of tumor in a remarkable way. Free DOX significantly suppressed the tumor growth comparing to that of control group and attained a final tumor volume of ~500 mm^3^. GNP on the other hand does not improve the antitumor effect to a great extent and the tumor volume grew linearly along with free DOX group. As expected, EGNP showed the maximum tumor suppression comparing to that of either free DOX or GNP. The final tumor volume of GNP and EGNP stood at 400 mm^3^ and 150 mm^3^, respectively. Throughout the study period, EGNP maintained a constant tumor volume and showed almost 90% tumor regression in this group. When tumor volumes are normalized, EGNP showed less than 2-fold increase in overall tumor volume which is significantly less than control group where final tumor volume increased up to 20-fold. Consistent with the maximum tumor regression in EGNP treated mice group, it showed a smallest tumor (~0.2g) while control group showed the largest tumor size (~0.8g) (Figure 6b). The tumor from free DOX and GNP groups were insignificantly different. This tendency agrees with our previous result that EGNP preferentially accumulated in the cancer cells than comparing to other formulations. Additionally, EGNP treated group remarkably maintained a higher lung concentration even after 24 h of administration of formulations. Results further indicate that cancer cells overexpressed with EGFR could be effectively recognized by EGF modified nanoparticles leading a high cellular accumulation in cancerous lungs [16].

Changes in body weight of mice were regarded as the indicator of safety profile. As seen from Figure 6c, none of the group showed any reduction in body weight indicating that DOX at a dose of 5 mg/kg was well tolerated. Furthermore, sustained release from nanocarriers also prevented the overexposure of drug to the normal cells. The slight increase in the body weight of control mice was attributed to the large tumor/tumor volume. The median survival time of EGNP administered group was 100% which is superior to any other groups (Figure 6d). As expected, untreated group showed lowest survival rate with only 20% of mice were alive at the end of study period. Similar trend was observed in DOX treated group. Such low survival rate could be due the toxicity as well as the inefficiency to control tumor growth.

### Histopathological and Immunohistochemical analysis

**Figure 7:**
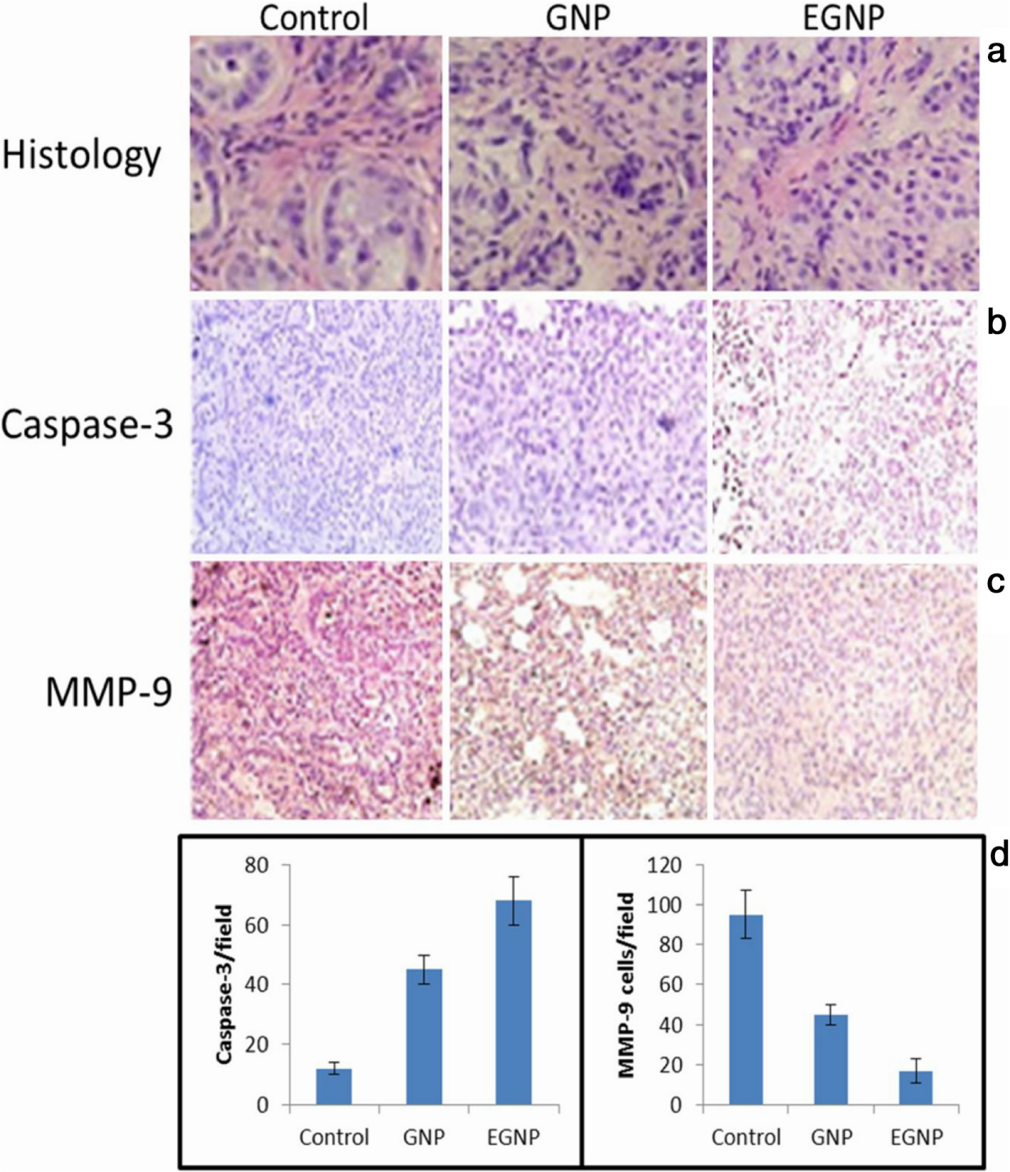
**Histopathological and Immunohistochemical analysis. (a)** Representative histopathological images of tumor sections **(b)** Immunohistochemical analysis of cleaved caspase 3 from tumor section collected from mice treated with GNP and EGNP **(c)** Immunohistochemical analysis of MMP-9 is presented **(d)** Quantitative IHC analysis of cleaved caspase 3 and MMP-9 is also presented.

H&E staining was performed on the respective tumors to observe the histological changes in the cancer cells. As can be seen (Figure 7a), control tumor exhibited a dense extracellular network which was in turn divided into the mini-compartments. Whereas, these extracellular networks were missing in GNP and EGNP treated group indicating its strong antitumor potential in the lungs.

Immunohistochemical analysis was performed to determine the apoptosis markers such as caspase-3 and MMP-9. IHC analysis of lung section showed that caspase-3 level significantly increased in formulation treated group comparing to that of control (Figure 7b). Specifically, EGNP showed 4-fold increase in caspase-3 than that of control group. Similarly, epithelial and mesenchymal transition (EMT) changes in tumor sections were carried out by western blot analysis. As is seen, inhalation of GNP and EGNP significantly reduced the expression of MMP-9 (Figure 7c). Especially, EGNP showed a 5-fold reduction in MMP-9 comparing to that of control group indicating its superior performance in the treatment of lung tumors. Overall, IHC analysis showed that elevated level of cleaved caspase-3 was in accordance with the superior anticancer efficacy following the inhalation of dosage forms for 3 weeks (Figure 7d). Furthermore, decreased MMP-9 levels reinforce the antiproliferative effects of DOX-loaded formulations.

Overall, our inhalation studies suggest a significant reduction in lung tumor weight in EGNP treated group compared to control group. The superior performance of EGF-modified nanocarriers in cancerous tissues was attributed to the EGFR receptor targeting (receptor-mediated interactions). Secondly, better intratumoral distribution restricted its entry into systemic circulation their by maximizing its anticancer effect on cancerous tissues. Thirdly, high intracellular accumulation for prolonged period of time (24 h) and sustained drug release pattern will allow a constant exposure of drug to the cancer cells. It has been reported that nearly 70% of inhaled particles deposit in the respiratory tract [24,25]. Therefore, a carefully designed delivery system by inhalation will greatly improve the chemotherapy efficiency. Importantly, EGF conjugation will greatly increase the specificity towards the lung cancer.

## Conclusion

In summary, we have successfully developed EGF-surface modified gelatin nanoparticles to target EGFR overexpressing lung cancers. The DOX-loaded nanoparticulate systems were administered by inhalation to increase the therapeutic effect of drug and to reduce its unwanted side effects. The EGNP showed significantly high intracellular accumulation in A549 and H226 cell lines via a receptor mediated endocytosis process. Consistently it showed a remarkable intratumoral distribution in experimental mice. GNP or EGNP did not activate the inflammatory response and had a low propensity to cause immune response indicating its high biocompatibility to lung tissues. EGNP treated group maintained a remarkably higher lung concentration even after 24 h of administration of formulations. The in vivo anticancer experiment showed that EGNP had a stronger tumor suppressive potential (90%) by comparison to free DOX and GNP treated group with 100% mice survival rate. IHC analysis of lung section showed that EGNP inhalation significantly increased the cleaved caspase-3 and MMP-9 levels reinforcing the strong antiproliferative effects of DOX-loaded formulation. Overall, results showed that EGF conjugation to nanocarriers could effectively target the EGFR overexpressing lung cancers when administered by inhalation. This ligand-targeted nanoparticulate system could be promising for the lung cancer treatment.

## Materials and methods

### Materials

Gelatin type A (porcine skin, bloom strength 175) was purchased from Sigma-Aldrich (China). Doxorubicin was procured from Afine Chemicals Limited, China. NeutrAvidin Fluorescein Conjugated (NeutrAvidin^FITC^), m-Maleimidobenzoyl-N-hydroxy-sulfosuccinimide ester (Sulfo-MBS), Sulfo-NHS-LC-biotin, 2-Iminothiolane.HCl, D-Salt™ Dextran desalting columns and EZ™ Biotin Quantization Kit were purchased from Pierce (Rockford, IL, USA). Human recombinant epidermal growth factor (EGF) was purchased from BioSource (Camarillo, CA, USA). All other chemicals were of reagent grade and used without any modifications.

### Methods

#### Preparation of doxorubicin-loaded gelatin nanoparticles

Gelatin nanoparticles (GN) were prepared as reported previously [17]. Briefly, GN was prepared employing a two-step desolvation method. 5 ml of 5% (w/v) of gelatin solution was mixed with 5 ml of acetone containing doxorubicin (10% w/w) and heated at 50°C. The precipitate was again re-dispersed at 50°C. Glutaraldehyde (0.25%) was added to cross-link the formed nanoparticle and stirred overnight at 1000 rpm. After evaporating residual acetone by vacuum drying, NPs were suspended in pure distilled water. The resulting NPs were stored at 4°C until further analysis or applications.

#### Preparation of EGF-surface modified gelatin nanoparticles

The surface modification of GN was done in two steps. Firstly, free amino group on the GN was converted to thiol groups by reacting with 2-iminothiolane. This thiol group was used to conjugate the respective functional group or ligand. For this, drug-loaded GN was dialyzed against sodium phosphate buffer containing 10 mM EDTA using a low molecular weight dialysis membrane. Following which, 2-iminothiolane was reacted with GN suspension for 2 h at 37°C to convert the amino group into thiol group. The resulting NP product was washed twice with sodium phosphate buffer containing 10 mM EDTA to remove the unreacted materials. Separately, NeutrAvidin (1 mg/ml) was mixed with Sulfo-MBS (2 mg) in 1 ml of sodium phosphate buffer (pH 7.2) and to activate the functional group. The activated NeutrAvidin was separated and purified using gel filtration column, followed by mixing with thiolated GN and left to react overnight at 4°C. The unbound biological moiety was removed by centrifugation using a centrifugal filter device. In the second step, surface modified NeutrAvidin was reacted with biotinylated EGF. For this, EGF was dissolved in PBS buffer and then biotinylation reagent was added and incubated for 1 h.

### Particle size distribution and zeta potential

The liposome solutions were suitably diluted to analyse the particle size distribution and zeta potential using dynamic light scattering (DLS) method. Malvern Zetasizer (Malvern, UK) was used determine the DLS characteristics. The samples were suitably diluted (200 μg/ml) with double distilled water such that mean count rate will be around 300 kcps. All measurements were performed at a fixed angle of 90° at 25°C room temperature. The results were expressed as the size ± SD.

### Loading efficiency

Loading efficiency was calculated from the total amount of drug added versus amount of drug entrapped in the nanoparticles. Briefly, drug-loaded complex was filtered by Amicon centrifugal filter by centrifuging at a high speed of 5000 rpm for 10 min. The filtrate was analyzed for unentrapped drug by spectrometric method at 254 nm. A standard curve of DOX was plotted.

$$ \mathrm{Loading}\ \mathrm{efficiency}\frac{\mathrm{Total}\ \mathrm{amount}\ \mathrm{of}\ \mathrm{D}\mathrm{O}\mathrm{X}\ \hbox{--}\ \mathrm{Amount}\ \mathrm{of}\ \mathrm{free}\ \mathrm{D}\mathrm{O}\mathrm{X} \times 100}{\mathrm{Total}\ \mathrm{weight}\ \mathrm{of}\ \mathrm{nanoparticles}} $$

### Morphology

The morphological examination of Lipo-DTX and Lipo-DTX/siRNA was carried out through a high resolution transmission electron microscopy (TEM, JEM-2010HR). Briefly, liquid sample was placed in a carbon coated copper grid and counter stained with phosphotungistic acid, followed by air drying for 2 h. The surface topography was further confirmed by the atomic force microscopy (AFM) where in samples were instilled on the mica surface and air dried for 2 h.

### In vitro release study

The release profile of DOX from GNP and EGNP was monitored by means of dialysis method. In this study, 1ml of NP dispersions was placed in the dialysis bag and both the borders were sealed with a dialysis clip. The dialysis bag (molecular cut-off of 10 kD) was incubated in 25 ml of release media (PBS, pH 7.4). The whole set up was placed in an automated shaker maintained at 100 rpm and 37°C. At predetermined time intervals, release media was collected and replaced with equal amount of fresh media. The released drug was quantified using a sophisticated spectrophotometer (Shimadzu UV Spectrometer, model mini1240) as mentioned above.

### Cytotoxicity assay

The A549 and H226 cells were cultured in normal RPMI media with 10% FBS and 100 units/mL penicillin, and 100 mg/ml streptomycin in 5% CO_2_ and 95% humidity atmosphere in humidifier. The cell viability/cytotoxic potential of individual formulation were performed by MTT assay. Briefly, cells were seeded into 96-well plate at a seeding density of 1 × 10^4^ cells in 0.1 ml growth medium and incubated for 24h. Following day, medium was removed and cells were incubated with various concentrations of free DOX, GNP and EGNP and incubated for 24 h. At each time intervals, cells were washed with PBS and treated with MTT solution (5 mg/ml in serum free media) and incubated for 3 h. The purple blue formazan crystals were extracted by the addition of DMSO. The optical density was measured at 570 nm on a microplate reader.

### Intracellular drug accumulation

The DOX accumulation in A549 and H226 cells were quantified by HPLC method. For this, both the cells were seeded in a 12-well culture plates at a density of 1 × 10^5^ per well and incubated 18 h. The attached cells were treated/exposed with free DOX, GNP, and EGNP and incubated for 30 and 120 min. The cells were treated at a DOX concentration of 10 μg/ml. Cells were washed twice with PBS and trypsinized. The trypsinized cells were centrifuged; cell pellets were treated with lysis buffer, and sonicated. The internalized drug was quantified by measuring the supernatant solutions via HPLC.

### Animals

Female, 6-week old, athymic Nu/nu mice were used to perform the antitumor efficacy study. The animal studies were approved by ‘Laboratory Animal Care’ and ‘Institutional Animal Ethics Committee’, SUN Yat-Sen University, China. The mice were caged in a pathogen free clean atmosphere and maintained in 12 h day/light conditions. Animals were maintained at standard conditions of 37°C and 60% humidity. Food and water were freely accessed to all the animal groups’ throughput the study period.

### Orthotopic tumor models

Prior to the cancer cell implantation, A-549 cells were cultured in F12K media containing 10% FBS and 1% penicillin-streptomycin antibiotic mixture. The orthotopic tumor models were developed by anesthetizing the mice with isoflurane, followed by a small skin incision was made on the left part of chest (5 mm below the scapula). A specialized Hamilton syringes with 28-gauge hypodermic needles were used to instil the cell suspensions into the left lung. At about 1.5 million A-549 cells (100 μl PBS) were quickly injected at a depth of 3mm and immediately syringe was removed. The wounds were surgically closed with the help of skin clips. The animals were kept under constant observation until the lung cancer develops. Respective formulations were administered by inhalation. The drug was administered at a dose of 5 mg/kg for 3 times at a gap of 3 days once. The lung weights and tumor volume were used for assessment of therapeutic activity of the treatments. At each time point, specified number of mice was sacrificed, and lung tumor was isolated. The tumor volume was measured using a Vernier calliper. Antitumor efficacy was calculated by plotting the tumor volume vs time for each formulation.

### Histopathology analysis

The tumor samples were analysed by histopathological staining. Briefly, 10 μm sections were deparaffinized and rehydrated, and stained with Hematoxylin and Eosin stain.

### Immunohistochemical analysis

The tumors were fixed with formalin and frozen until further use. The samples were rehydrated by immersing in the graded alcohol and embedded in paraffin. The paraffin sections were cut into 10 μm thin sections and mounted on a poly-L-lysine-coated slide. It was deparaffinized and rehydrated through graded alcohol and incubated for 20 min with 3% hydrogen peroxide. Antigen retrieval for cleaved caspase-3 and MMP-9 staining was done with sodium citrate buffer for 10 min, followed by heated at 95°C and cooled thereafter. Protein block solution was used for 1h, followed by samples were stained and stained with caspase-3 and MMP-9 antibodies. The samples were exposed to substrate-chromogen to develop the color.

### In vivo biocompatibility of nanoparticles

Six to eight week old female Balb/c mice was selected to carry out the in vivo experiment. Firstly, mice were treated with 50 μl of 1 mg/ml of respective GNP and EGNP dispersions. Lipopolysaccharide was used as a positive control which can induce acute lung injury. The mice were sacrificed at day 1, 4, and 7 post-treatment and lungs were surgically removed. The lung tissues were homogenized and centrifuged and the supernatant solution was deproteinated. The glutathione levels in the supernatant were evaluated by glutathione assay kit as per the instructions. IL-6 levels were determined from the BAL fluid. The trachea was exposed from the mice sacrificed at respective days. 1 ml of cold saline was injected and immediately pulled back and collected. The cellular materials were removed by centrifugation and the supernatant was subjected to IL-6 assay by the IL-6 ELISA kit.

### In vivo deposition of doxorubicin

Orthotropic tumor models were developed as mentioned above. The tumor mice were exposed to aerosol. A nebulizer (AP-100100, APEX Medical Corp., Taiwan) was used to generate aerosol particles. The free DOX, GNP, and EGNP suspensions were used for the inhalation. The DOX concentration deposited in the lung from different formulations were measured. For this, mice were sacrificed at 0.5, 12, and 24 h post-treatment. The lung was carefully removed and washed twice with PBS and soaked in 70% nitric acid for 12-15 h. The organs were digested at 90°C for 2 h. The organs were then homogenized, centrifuged, supernatant was collected, and analyzed using HPLC method.

### Statistical analysis

All data are expressed as mean ± standard deviation. Statistical differences between groups were tested using one-way analysis of variance (ANOVA). Statistical significance was set in advance to a probability level of 0.05.

## Abbreviations

EGF: Epidermal growth factor
EGFR: Epidermal growth factor receptor
EGNP: EGF-conjugated gelatin nanoparticles
GNP: Gelatin nanoparticles
DOX: Doxorubicin
IL6: Interleukin-6
LPS: Lipopolysaccharide
NSCLC: Non-small cell lung cancer
BAL: Bronchoalveolar lavage
